# Utilization of bar and *izakaya*-pub establishments among middle-aged and elderly Japanese men to mitigate stress

**DOI:** 10.1186/1471-2458-12-446

**Published:** 2012-06-18

**Authors:** Mayumi Ohnishi, Rieko Nakao, Ryokko Kawasaki, Akiko Nitta, Yukari Hamada, Hideyuki Nakane

**Affiliations:** 1 Unit of Rehabilitation Sciences, Nagasaki University Graduate School of Biomedical Sciences, Nagasaki, Japan; 2 Unit of Nursing, Nagasaki University Graduate School of Biomedical Sciences, Nagasaki, Japan; 3 Ken’ou Public Health Office, Nagasaki, Japan

**Keywords:** Bar and *izakaya*-pub, Middle-aged and elderly men, Mitigation of stress, Prevention of suicide

## Abstract

Japanese suicide rate is one of the highest among industrialized nations, especially following the economic crisis of the 1990s, with more than 30000 suicides every year since 1998. Previous studies have pointed out to relationships between overwork and/or job stress, and death and other health risks, and suggested several possible avenues for releasing stress and emotional burden, including suicidal ideation, through talking with intimate friends, family, and specialists, such as counselors and physicians. The present study was performed to explore the potential role of owners and managers of bars and *izakaya*-pub establishments in mitigating stress of middle-aged and elderly Japanese men by having informal conversations with them. A self-administered questionnaire was posted to all bars and *izakaya*-pubs registered in Ohmura-city, Nagasaki prefecture, in December 2009. Among 260 bars and *izakaya*-pubs, a total of 103 owners and managers completed the questionnaire. More than half of the respondents experienced engaging in conversations with their customers regarding customers’ various personal and private issues. The most frequently talked about problem was that regarding work (56.3%). Regardless of sex and age of the respondents, those with longer working experience in bar and *izakaya*-pub establishments were more likely to have had customers confiding in them financial problems including debts/loans (adjusted odds ratio: 5.48, *p* = 0.033). Owners and managers of bars and *izakaya*-pubs may be in a position to act as “listeners”, to whom middle-aged and elderly men can talk about their personal problems casually and without having to worry about conflict of interests, and direct those in need to professional counseling.

## Background

Among the countries included in the Organization for Economic Cooperation and Development, Japan shows a high prevalence rate of deaths due to suicide, especially following the economic crisis of the 1990s, with more than 30000 suicides every year since 1998. Among these suicides, 51.3% occurred among middle-aged and elderly men aged 40 years old and above in 2010 [1]. Furthermore, it has been reported that about 10% of rural residents aged 40 years old or above in Japan have had suicidal ideation, and about 18% have had suicidal ideation sometime in their life [2]. In another study performed among elderly Japanese men and women aged over 65, 12.3% of the respondents reported having had thoughts about death or suicide [3]. A more recent study by Kaneko and colleagues has also reported that 26.3% of elderly Japanese men and women had experience of suicide ideation [4]. Suicide rate among the middle-aged working Japanese men has also been increasing since the late 1990s [5].

According to a study of background factors associated with suicide in Japan, some of the possible causes behind suicide included bullying, truancy, overwork, and debts/loans, including debts due to gambling [6]. Among 33334 suicide deaths in 2010, the National Police Agency of Japan reported 22.3% were related to economic problems, with 19.6% due to debts/loans, including multiple debts. It is also generally known that mental disorders increase the risk of committing suicide and, in a study conducted by Harris and Barraclough, were associated with excess death among the mentally ill [7]. In Japan too, the National Institute of Mental Health of Japan has in the past reported possible associations between suicide deaths and depression and/or alcohol-related problems [8]. In view of this situation, the Japanese Ministry of Health, Labour, and Welfare, local governments and relevant organizations began implementing programs and activities to prevent suicide and promote mental health, especially among middle-aged men, from early 2000s.

Many studies have also indicated relationships between overwork and/or job-related stress and death and/or health risks [9-13]. For example, studies from other industrialized countries have showed that negative work characteristics, can become a risk factor for depressive symptoms in men [14]. Depressive symptoms were associated to problem drinking, and problem drinking was associated with suicidal ideation [15]. Aside from mental illness, unemployment has also been pointed as being possibly associated with increased risk of suicide. [16] In Japan, several studies have indicated that low levels of social support along with long working hours and heavy workloads could be responsible for depression and suicide among men [17,18]. A perceived lack of understanding from others with regard to health is also associated with an increase in rate of depressive symptoms [19].

On the other hand, several possible avenues for releasing stress and emotional burden, including suicidal ideation, have been suggested in various studies, including talking with intimate friends, family members, and specialists, such as counselors and physicians [2,3]. A study by Ono and colleagues in Japan found out that one third of people who reported having had thoughts about death or suicide have consulted other people about their problems, including family members and professionals [3]. A lack of a social support network among elderly Japanese men and women is associated with thoughts of suicide [20]. Japanese employees with job stress may protect their mental health status by their high sense of coherence, and social support may also improve mental well-being [9]. Seeking professional counseling may appear the most effective means of releasing stress and preventing suicide, however, the previous studies have also shown that men in general are also more reluctant to seek professional psychological counseling, [21] and also that those with suicidal ideation and who have attempted to commit suicide do not seek help [22]. In Japan too, the authors, as mental health counselor at clinical settings and/or public health office, have observed that Japanese middle-aged and elderly men may feel more comfortable seeking informal assistance, such as talking with an outsider (a third party), as they prefer to guard themselves against losing face in front of people whom they know and with whom they wish to maintain dignity.

Another means of informal work stress mitigation is drinking alcohol. Several studies in Japan have reported associations between job stress and drinking behavior [13,23]. On the one hand, stress-related drinking causes negative impact on health, such as alcohol dependence and liver dysfunction. For example, heavy drinking has been related to occupational stress, including inappropriate supervision, intragroup conflict, cognitive demands, quantitative workload, and underutilization of abilities among different age groups in Japan [24]. Kawakami *et al.* have also reported that overtime, job overload, and poor intrinsic work rewards were associated with drinking problems among working men [13]. On the other hand, alcohol drinking can also bring positive outcome. “Nominication”, which is a Japanese word consisting of “*nomi*” meaning “drinking” in Japanese, and “communication”, is an important aspect of work culture in Japan and refers to communicating with work colleagues and business associates over drinking sessions. “Nominication” may not only facilitate smooth business but also allows workers to talk about issues which may be considered too private or too serious to mention while sober, and ameliorate job dissatisfaction [23]. In a separate study, it has been shown that alcohol drinkers demonstrated higher subjective health status than non-drinkers [25]. In an unpublished study by the same authors of the present study performed in Nagasaki Prefecture, which was conducted among 816 randomly selected Japanese men 40 years old and over, 48% of respondents reported drinking alcohol every day, while 23% reported drinking alcohol sometimes. The average alcohol intake per day was less than 1.5 units. Among the respondents, 35% considered alcohol drinking as a means of alleviating stress, and 9% reported having discussed their personal/private problems such as debts/loans, family issues, and others, about which they were less likely to consult friends, colleagues and family members, with the owners/managers of a “snack bar” and/or *izakaya*-pubs.

It may be useful here to distinguish between “bars” and “*izakaya*-pubs”, for readers unfamiliar to drinking culture of Japan. “Bars” in Japan refers to an alcohol-serving bar, usually with Karaoke, and the owner and the manager of a bar may either be the same person or, the manager maybe hired separately by the owner. Bars are usually relatively small, customers can sit and talk closely with the staff. Customers of such bars are usually middle-aged and elderly men, and their main objective is to drink alcohol. *Izakaya*-pubs on the other hand are casual Japanese-style pubs which serve various small dishes which go well with alcohol. Customers of *izakaya*-pubs are both male and female, from various age groups and professional backgrounds.

Based on the observation that firstly, Japanese middle-aged and elderly men may feel more comfortable talking about their personal issues with a third party in an informal setting, and secondly, that some men were utilizing bars and *iazakaya*-pubs as places to talk about their personal problems, we thus conducted this study to investigate whether or not middle-aged and elderly Japanese men sought to release their stress and emotional burden by talking to owners/managers of such establishments about their private problems.

## Methods

### Study participants and area

Nagasaki prefecture is one of the provincial prefectures in south Japan, and has a moderate prevalence rate of suicide-related deaths. Omura-city is within the catchment area of Kenou Public Health Office, which is the principal public health office in Nagasaki prefecture. A total of 260 bar and *izakaya*-pub establishments (183 bars and 77 *izakaya*-pubs) were registered in the catchment area of Kenou Public Health Office as of December 2009.

### Study methodology

A self-administered questionnaire was posted to all bars and *izakaya*-pubs registered within the catchment area of Kenou Public Health Office in December 2009. Respondents were either bar owners, who also served customers themselves, or managers, who were employed by owners to oversee actual hospitality services. The completed questionnaires were returned to Kenou Public Health Office by the end of January 2010. The study team performed telephone follow-up with bars and *izakaya*-pubs that had not returned the questionnaires in February 2010, and performed telephone interviews using the same questionnaire if the subjects agreed to participate in the study.

The questionnaire elicited basic demographic information of the participants, and years of experience of working in bars and *izakaya*-pubs. The participants were also asked about their experiences of engaging in conversations with their customers aged ≥ 40 years old, contents of such conversations (i.e.personal/private issues), and any difficulties they experienced in engaging in such conversations (Table 1). The contents of conversations regarding personal/private issues were classified into several categories including work related problems, partner/family, children, disease, debt/loans, love, nursing care of family, and others, and the participants were asked as a multiple-choice question. In addition, free space was provided to allow the respondents to write any other thoughts and opinions regarding their experience of serving customers. In establishments with both owners and managers, those who were responsible for directly serving the customers were requested to complete the questionnaire.

**Table 1:** **Questions to bar and*izakaya*-pub regarding experiences of serious conversation with customers**

· Have you ever experienced having conversations regarding personal/private issues with customers? (Yes, No)	
· What was the content of such conversations regarding personal/private issues with customers? (multiple choice)	
1) Work related problems	
2) Disease	
3) Debt/loan	
4) Partner/Family	
5) Children	
6) Love	
7) Nursing care of family	
· Have you ever experienced having serious conversations with customers? (Yes, No)	
· Have you ever experienced being unable to handle a serious conversation, including suicide ideation, from a customer? (Yes, No)	
· What responses have you given regarding serious conversation with customers? (multiple choice)	
1) Listen	
2) Give advice	
3) Provide encouragement	
4) Inform professional/special consultation/institution	
5) Other (specify: )	
·To what points do you pay attention when listening to customers? (multiple choice)	
1) Take care of confidentiality regarding conversations with customers.	
2) Listen to the end of the customer’s talk.	
3) Encourage and cheer the customer up.	
4) Tell the customer they can talk to you any time.	
5) Inform professional/special consultation/institution.	
6) Others (specify: )	
Please give details regarding anything else about customer services including conversation with customers that you would like to mention. (A free writing space is provided.)	

In the analysis, variables including experiences of conversation with customers and the contents of conversations were compared between respondents from bars and from *Izakaya*-pubs. The rationale behind this comparison was based on the hypothesis that owners and managers of bars were more likely to experience holding intimate conversation with their customers because of the smaller size of the establishment, which makes interpersonal distance between the customer and the bar owner/manager closer, and also because the main objective of the customers of bars is to drink alcohol.

### Statistical analysis

The distributions of demographic characteristics, type of business, and number of years of business experience were calculated. The chi-square test was used to evaluate the significance of associations among experiences of being confided in by customers, type of conversation, and demographic characteristics. Logistic regression analysis was performed to assess the associations between demographic characteristics and type of conversation. In all analyses, *p* < 0.05 was taken to indicate statistical significance. SPSS Statistics 19 was used for the analysis.

### Ethical considerations

The study protocol was approved by the Ethics Committee of the Nagasaki University Graduate School of Biomedical Sciences. A written explanation regarding study participation, including ethical considerations, was provided to each of the study participants along with the questionnaire. Informed consent from the study participants was provided by returned completed questionnaire to the study team.

## Results

Completed questionnaires were returned from a total of 84 study participants, including 56 bars and 28 *izakaya*-pubs. In addition, 19 participants, including 7 bars and 12 *izakaya*-pubs, participated in the study by telephone interview with the study team. A total of 103 respondents (63 bars and 40 *izakaya*-pubs) were thus included in the present study, and the response rate was 39.6% (34.6% from bars and 51.9% from *izakaya*-pubs).

Table 2 shows the demographic characteristics and number of years of experience in business related to bar and/or *izakaya*-pub establishments of the respondents. Most of the respondents from *bars* were female (76.2%), while most of the respondents from *izakaya*-pubs were male (82.5%) (*p* < 0.001). About half (52.4%) of the respondents from bars were 60 years old or older, while the majority of the respondents from *izakaya*-pubs were aged below 60 years old (75.0%) (*p* = 0.006). The majority of the respondents from both types of establishments reported having more than 11 years of experience related to working in bars and/or *izakaya*-pubs (84.1% and 75.0%, respectively). However, only 42.5% and 68.3% of the respondents from bars and *izakaya*-pubs had worked at the same establishment for more than 11 years, respectively (*p* < 0.05).

**Table 2:** **Demographic characteristics of respondents (*n*** **= 103)**

	**Bar (*n* = 63)**		***Izakaya*-pub (*n* = 40)**		***p***
	***n***	**%**	***n***	**%**	
Sex					
Male	15	23.8	33	82.5	< 0.001
Female	48	76.2	7	17.5	
Age group					
< 60 years old	30	47.6	30	75.0	0.006
≥ 60 years old	33	52.4	10	25.0	
Years of experience					
≤ 10 years	10	15.9	10	25.0	0.254
≥ 11 years	53	84.1	30	75.0	
Years of business with bar or *izakaya*-pub					
≤ 10 years	20	31.7	23	57.5	0.010
≥ 11 years	43	68.3	17	42.5	

(chi-square test).

A total of 70 respondents (68.0%), corresponding to more than half of those from both bars (69.8%) and *izakaya-*pubs (65.0%), reported having conversations regarding personal/private issues with their customers, but there were no significant relations among demographic characteristics and number of years of working experience by chi-square test (Table 3). Seventeen respondents (16.5%) evaluated their conversation with the customers as being serious, and only 7 respondents (6.5%) reported difficulty in engaging in such serious conversations.

**Table 3:** **Experience of serious conversation with customers (*n*** **= 103)**

	***n***	**Experience of conversation regarding personal/private issues (%)**	**Experience of serious conversation (%)**	**Experience of consultation that was subjectively difficult to deal with (%)**
Type of business				
Bar	63	69.8	15.9	6.3
*Izakaya*-pub	40	65.0	17.5	7.5
Sex				
Male	48	64.6	20.8	8.3
Female	55	70.9	12.7	5.5
Age group				
< 60 years old	60	61.7	16.7	8.3
≥ 60 years old	43	76.7	16.3	4.7
Years of experience				
≤ 10 years	20	70.0	20.0	10.0
≥ 11 years	83	67.5	15.7	6.0
Years of business with *izakaya* pub or bar				
≤ 10 years	43	62.8	18.6	11.6
≥ 11 years	60	71.7	15.0	3.3

As shown in Table 4, the types of conversation with customers were associated with demographic characteristics of the respondents. The most frequent type of conversation was that regarding work (*n* = 58, 56.3%), followed by partner/family (*n* = 38, 36.9%), children (*n* = 37, 35.9%), disease, debts/loans, love (all *n* = 34, 33.0%), and nursing care of family (*n* = 28, 27.2%). Respondents from *izakaya*-pubs were more likely to have experienced conversation with customers about “children” (*p* = 0.007) and “nursing care of family” (*p* = 0.027). Older respondents were more likely to have experienced conversations regarding “disease” (*p* = 0.041) and “nursing care of family” (*p* = 0.017). Respondents who had more than 11 years of working experience were more likely to have experienced conversations about “debts/loans” (*p* = 0.015). Although there were no statistical significant differences, male owner/manager experienced to have conversation regarding “debt/loan” and “love”, and female owner/manager experienced to have conversation regarding “family/partner” and “nursing care of family”. A total of 78 respondents (75.7%) reported that they were conscious about protecting confidentiality of the conversations they had with their customers (data not shown).

**Table 4:** **Contents of personal/private conversation by customers depends on demographic characteristics of respondents (*n*** **= 103)**

	***n***	**Work related problems**		**Disease**		**Debt/loan**		**Partner/family**		**Children**		**Love**		**Nursing care of family**	
		**%**	***p***	**%**	***p***	**%**	***p***	**%**	***p***	**%**	***p***	**%**	***p***	**%**	***p***
Type of business															
Bar	63	50.0	0.304	13.0	0.605	37.5	0.440	30.0	0.248	20.0	0.007	35.0	0.732	15.0	0.027
*Izakaya*-pub	40	60.3		34.9		30.2		41.3		46.0		31.7		34.9	
Sex															
Male	48	54.2	0.682	31.3	0.723	39.6	0.185	31.3	0.267	29.2	0.182	37.5	0.365	18.8	0.072
Female	55	58.2		34.5		27.3		41.8		41.8		29.1		34.5	
Age group															
< 60 years old	60	50.0	0.127	25.0	0.041	28.3	0.233	35.0	0.638	28.3	0.058	33.3	0.934	18.3	0.017
≥ 60 years old	43	65.1		44.2		39.5		39.5		46.5		32.6		39.5	
Years of experience															
≤ 10 years	20	55.0	0.895	25.0	0.396	10.0	0.015	50.0	0.176	20.0	0.098	30.0	0.750	20.0	0.421
≥ 11 years	83	56.5		34.9		38.6		33.7		39.8		33.7		28.9	
Years of business with *izakaya*-pub or bar															
≤ 10 years	43	48.8	0.195	25.6	0.175	24.4	0.075	39.5	0.638	27.9	0.151	30.2	0.612	23.3	0.448
≥ 11 years	60	61.7		38.3		39.7		35.0		41.7		35.0		30.0	

(Chi-square test).

As shown in Table 5, regardless of sex, age, and type of establishment, the respondents who had more than 11 years of working experience were more likely to have experienced conversations about “debts/loans” (adjusted odds ratio (AOR): 5.48, 95% confidence interval (CI): 1.15, 26.08, *p* = 0.033). After controlling for sex, age, and number of years of working experience, the respondents from bars were less likely to have experienced conversations regarding “children” (AOR: 0.30, 95%CI: 0.09, 0.93, *p* = 0.038).

**Table 5:** **Associations between demographic characteristics and type of serious conversation from customers (*n*** **= 103)**

	**Experience of consultation**			**Experience of serious consultation**			**Experience of consultation that was difficult to deal with**			**Work related problems**			**Disease**		
	**AOR**	**95% CI**	***p***	**AOR**	**95% CI**	***p***	**AOR**	**95% CI**	***p***	**AOR**	**95% CI**	***p***	**AOR**	**95% CI**	***p***
Type of business															
Bar	1			1			1			1			1		
*Izakaya*-pub	1.02	0.35, 2.92	0.975	0.73	0.20, 2.60	0.626	0.82	0.13, 5.07	0.831	0.67	0.25, 1.82	0.429	0.97	0.33, 2.88	0.955
Sex															
Male	1			1			1			1			1		
Female	1.12	0.40, 3.15	0.837	0.45	0.13, 1.61	0.219	0.65	0.10, 4.01	0.643	0.80	0.30, 2.14	0.655	0.89	0.31, 2.57	0.829
Age group															
< 60 years old	1			1			1			1			1		
≥ 60 years old	2.11	0.82, 5.41	0.120	1.20	0.38, 3.76	0.759	0.63	0.10, 3.76	0.608	1.83	0.77, 4.35	0.170	2.34	0.95, 5.77	0.065
Years of experience															
≤ 10 years	1			1			1			1			1		
≥ 11 years	0.73	2.44, 2.19	0.574	0.71	0.20, 2.59	0.605	0.64	0.11, 3.76	0.625	0.87	0.31, 2.41	0.789	1.28	0.41, 4.07	0.671
	**Debt/loan**			**Partner/family**			**Children**			**Love**			**Nursing care of family**		
	**AOR**	**95% CI**	***p***	**AOR**	**95% CI**	***p***	**AOR**	**95% CI**	***p***	**AOR**	**95% CI**	***p***	**AOR**	**95% CI**	***p***
Type of business															
Bar	1			1			1			1			1		
*Izakaya*-pub	1.29	0.41, 4.07	0.669	0.66	0.23, 1.87	0.437	0.30	0.09, 0.93	0.038	0.91	0.32, 2.59	0.865	0.46	0.16, 1.58	0.219
Sex															
Male	1			1			1			1			1		
Female	0.51	0.16, 1.58	0.241	1.25	0.46, 3.39	0.665	0.78	0.27, 2.28	0.646	0.64	0.23, 1.87	0.393	1.23	0.40, 3.80	0.725
Age group															
< 60 years old	1			1			1			1			1		
≥ 60 years old	1.83	0.71, 4.70	0.209	1.20	0.50, 2.91	0.683	1.58	0.64, 3.92	0.323	1.04	0.42, 2.56	0.932	2.29	0.87, 6.01	0.093
Years of experience															
≤ 10 years	1			1			1			1			1		
≥ 11 years	5.48	1.15, 26.08	0.033	0.45	0.16, 1.25	0.126	2.10	0.61, 7.26	0.242	1.19	0.40, 3.53	0.758	1.11	0.31, 3.95	0.871

(Logistic regression analysis).

In the free space, some respondents described the contents of the conversations in detail. For example, one respondent described one of his customers “wanting to commit suicide, because of family discord and debt”, while another respondent wrote about his customer “regretting to accept becoming a guarantor.” There were also those wrote that they wished to have information and knowledge regarding professional counseling services, so that they could refer their customers whom they suspected as being depressed and in need of medical help.

## Discussion

The results showed that consistent with our hypothesis, the manager and owners of bars and *izakaya*-pubs who participated in the present study reported having had conversations regarding personal/private issues, including serious problems and concerns, with their customers. They also felt that by holding such conversations, they provided emotional care and helped the customers to relieve stress.

Past studies have indicated that while poor mental health and family problems are possible factors which hinder access to consultation at primary care level, [26] being married showed protective effect against suicide regardless of socioeconomic inequality [27]. Relatives and friends also played key roles in encouraging suicidal individuals to seek help and a range of lay interventions, including non-medical help-seeking, have been identified among suicide victims [28]. Help-seeking behaviors in times of crisis and access to effective treatment could also reduce risks of committing suicide [29]. However, in cultures where such family and partner function cannot be expected to function, it becomes necessary to explore other alternatives. In Japan, even when they believe they have good relationship with their wives or other family members, men tend not to talk about serious problems, such as business failure and debts due to gambling and/or other reasons to which negative social stigma is attached, at home, because of their wish to maintain dignity and avoid losing face. They often also prefer not to talk about work-related problems with colleagues or their supervisors/as they tend to fear that such issues will be reflected in their performance evaluation.

Our results showed that owners and managers of bars and *izakaya*-pubs who participated in the present study had experiences of having conversations regarding personal/private issues, including serious problems, with their customers. There are several possible reasons why bars and *izakaya*-pubs may become the ideal place for Japanese men to unburden their troubles. For one, as noted earlier, because of their relatively small spatial size and close distance between the customer and the owners/managers, bars and *izakaya*-pubs easily facilitate intimate conversations. Since the main objective of the customers is to drink, they are usually at ease and “talking” becomes the natural act, and not something they are formally asked to do. As there is no perceived conflict of interest, the customers may talk about anything, from family problems to work and financial troubles. Our results have also indicate, respondents with longer years of working in the business tended to experience customers confiding in them about debt and loans, while female respondents tended to experience listening to customers talk about family and relationship problems. Furthermore, since such drinking activity is already settled as a cultural part of everyday lives of Japanese men, access is much easier than compared to, for example, professional counseling services or other medical institutions.

Bar and *izakaya*-pub owners and managers are not specialists in solving such problems and it is unrealistic to expect them to take on the role of professional counselors. However, our results pointed to the possibility of them playing a key role in referring their customers, whom they judge as requiring professional help, to appropriate counseling and other relevant services. For example, they may be offered seminars on mental health, alcoholism and basic conversation skills with people suspected of having depressive symptoms. Some of the respondents in the present study reported that they encouraged their customers who were depressed, however, as is well-know, encouragement is often not a suitable reaction to depressed individuals. Had they known this and received appropriate training, they may have been able to respond differently.

Bar and *izakaya*-pub owners and managers may also be informed about the role of public health centers. Although it is often the case that Japanese men, if they were to seek care because of their depressive symptoms, firstly consult primary health care physicians, assessment can sometimes be crucially inadequate because of lack of knowledge about mental health among the physicians [30,31]. Certainly, it is important to promote knowledge and understanding of mental health among the physicians. However, sometimes people suffering from depression may receive the care they need faster by being referred to their local public health center.

Furthermore, local governments may involve such establishments in suicide prevention activities by, for example, distributing educational pamphlets and information regarding how and where to seek help through bars and *izakaya*-pubs, thereby further enhance help-seeking behaviors.

Finally, it has been said that knowledge of and attitudes toward suicide and depression are correlated with suicide rate [6]. It may thus be necessary to improve the mental health literacy of the target population and enable them to seek help when they most need it. Kaneko and Motohashi have reported that poor mental health literacy was a possible factor contributing to male vulnerability to suicide, [32] and other studies indicated a significant reduction in suicide rate after implementation of community-based interventions by health promotion approach [33,34]. For example, participation in mental health workshops facilitated conversation with specialists regarding depression or suicidal ideation and improved access to counseling [35]. At primary care level, an approach using educational pamphlet about depression and suicide contributed to an increase in willingness to confide in clinicians, friends, and spouses among people feeling depressed or with suicidal ideation [33]. Interventions directed toward company employees, especially small company/factory employees, have also been suggested to increase their mental health literacy [30]. Skills training for school staff serving as natural gatekeepers who facilitated appropriate help-seeking demonstrated positive impact on increasing students’ help-seeking behaviors [36].

The response rate was too low to conduct detailed statistical analysis in this study, and it may reflect on the results as selection bias and reporting bias, which are under or over estimations of experiences regarding conversation of private issues between owners or managers and their customers. One possible reason for the low response is that both bars and *izakaya*-pubs are subject to hygiene inspections by public health offices. Thus, even though the section responsible for such inspections and the section involved in the present study are different, managers and/or owners of such establishments may have felt awkward or annoyed in responding. Or, they may simply have had no time, or not have been interested or motivated in the topic of the questionnaire if they had no experience of participating in serious conversations with their customers. No comparisons were performed with other cities or regions with different socioeconomic characteristics, and therefore the results cannot be generalized to other areas. The present study included no discussion of the relationships between alcohol consumption and depression and/or suicide. Further studies are required to evaluate the associations between how drinking alcohol facilitates conversation and outcomes of conversations in bar and *izakaya*-pub establishments, such as the effects on resolution of customers’ problems and customers’ health status. In addition, future assessments of the relations between alcoholism and frequenting bars and/or *izakaya*-pubs are necessary to determine the possible negative impacts of alcohol drink on customers’ mental health status. The present study also did not directly target the customers of bar and *izakaya*-pub establishments. Therefore, it was not possible to discuss the detailed effects and outcomes on the customers after conversation with owners/managers, e.g., how many people avoided suicide, and the customers’ intention to talk and consult with a professional regarding their personal/private problems. These points should also be investigated in future studies.

Despite these limitations, the study results indicated a potential for managers and owners of bars and *izakaya*-pubs, especially those with longer years of working experience, to play a role in assisting middle-aged and elderly men to seek professional mental care, by providing appropriate seminar and workshops on mental health, and building partnership with local governments and professional organizations.

## Competing interest

The authors declare that they have no competing interests.

## Authors’ contributions

MO was responsible for data analysis and for writing the manuscript. RN, RK and AN contributed to conceptualization of the study, data analysis, interpretations of the results and revisions of the draft manuscript. YH prepared the draft of the study proposal, and was responsible for data collection, and contributed to the writing of the manuscript. HN supervised conceptualization of the analytical framework, data analysis and writing of the manuscript. All authors read and approved the final manuscript.

## Pre-publication history

The pre-publication history for this paper can be accessed here:

http://www.biomedcentral.com/1471-2458/12/446/prepub
